# COVID-19 impact on reproduction and fertility

**DOI:** 10.5935/1518-0557.20200103

**Published:** 2021

**Authors:** Akash S Mali, Madhuri Magdum, Jiri Novotny

**Affiliations:** 1 Department of Physiology, Faculty of Science, Charles University, Prague Czech Republic; 2 Department of Hospital Pharmacy, Samata Hospital, Dombiwali (MH), India

**Keywords:** COVID-19, SARS-CoV-2, Fertility, Reproduction

## Abstract

The COVID-19 pandemic is an unexpected worldwide situation, and all countries have implemented their own policies to curb the spread of the virus. The pathophysiology of COVID-19 has opened numerous hypotheses of functional alterations in different physiological aspects. The direct impact of SARS-CoV-2 on the urogenital organs of males and females is still to be assessed. Nevertheless, based on biological similarities between SARS-CoV and SARS-CoV-2, several hypotheses have been proposed. In this study, we will discuss the possible mechanism of action, and potential effects on the male/female reproductive system and fertility.

## Introduction

*Coronaviridae* is a large family of enveloped, single-stranded positive RNA viruses, known to infect not only Bats and snakes, but also other mammals including humans, mainly causing respiratory, gastrointestinal and neurologic diseases (Mungroo *et al*., 2020). The SARS-CoV-2 genome analysis found sequence similarities between SARS-CoV and MERS-CoV to be 79.5% and 50%, respectively (Jin *et al*., 2020). The first case was transmitted from animal to human (December 2019 in Wuhan, China and named as COVID-19) and today, SARS-COV-2 affects 213 countries and territories around the world (total infected population is 92,088,149; with 1,972,267 reported deaths). SARS-CoV-2 has four main structural proteins, specifically spike (S), membrane (M), small membrane (SM) proteins and nucleocapsid (N). The S protein is essential for the virus to fuse to the host cell through the receptor-binding-domain (Monteleone *et al*., 2020). The key pathway for SARS-CoV-2 to entry the cell is through the S-protein attachment to the angiotensin-converting enzyme 2 (ACE2), which is highly expressed in spermatogonial stem cells, seminiferous duct cells, type II alveolar cells, myocardial cells, bladder urothelial cells and proximal tubule cells. The virus transmission in humans occurs via respiratory droplets from sneezing and coughing, the foremost vehicle for its spread. Recently, the WHO reported the possibility of airborne transmission, as well as the presence of RNA in urine and semen, increasing the possibility of sexual transmission.

A recent report has proposed that the SARS-CoV-2 virus critically depends on the angiotensin-converting enzyme 2 (ACE2) and the transmembrane serine protease (TMPRSS2 and TMPRSS4), as a receptor to enter the cells (Lu *et al*., 2020; Stopsack *et al*., 2020), which is comparable to the mechanism through which the SARS-CoV penetrates cells. The EC domain of the ACE2 acts as a cell surface receptor for the S-domain on the SARS-CoV-2 envelope. Viral glycoproteins comprise an extracellular domain, a TM (transmembrane) domain and an IC domain. An S1 unit, which bonds to the angiotensin-converting enzyme-2 peptidase domain (PD) via the receptor-binding domain (RBD), shapes the extracellular domain; a second S2 unit facilitates membrane fusion instantaneously, binding to the viral receptor. Dimitrov *et al*. (2003) reported angiotensin I breakdown into angiotensin (1-9), through a PD domain and transformed into angiotensin (1-7), by the angiotensin-converting enzyme. The angiotensin-converting enzyme 2 directly converts angiotensin II into angiotensin (1-7). The ART1 receptor binds to the angiotensin II, resulting in inflammation and fibrosis. To protect against organ damage, RAS (renin angiotensin system) activation is inhibited by ACE2. In the SARS-CoV-2 infection process, the ACE2 receptors are saturated by binding with the viral envelope, activating angiotensin II, which cannot be converted. The additional angiotensin II explain the pulmonary symptoms that are typical of SARS-CoV-2. Angiotensin (1-7) binds to the ART2 and mitochondrial assembly (MAS) receptors. There is confirmation of the presence of ACE2, angiotensin (1-7) and its MAS receptors in the testicles, specifically in the Sertoli and Leydig cells (Gianzo *et al*.,2018). The key function of the Leydig cell is to generate sex steroid hormones, especially testosterone. Per se, the MAS receptors suggest that angiotensin (1-7) modulates the secretion of testosterone. The presence of angiotensin (1-7) and MAS receptors in the seminiferous tubules might elucidate the involvement of Sertoli cells and germinal cells (Reis *et al*., 2011). However, the testicular expression of ACE2 may determine the possible entry of the virus into the testicles, although Ding *et al*. (2004), reported direct infection in other organs, but not in the testicles. Currently, there is no information of the possible entry of SARS-CoV-2 in testicles through ACE2 or other mechanisms. Ma *et al*. (2020) showed significantly higher levels of prolactin and serum luteinizing hormone (LH) in covid-19 patients than in healthy men, but no significant changes in serum testosterone levels. This result indicates that the early stage of infection impaired testosterone production and stimulated LH release, which temporarily maintained the level of testosterone. Wang *et al.* (2020) concluded that the testis is a high-risk organ, very much susceptible to COVID-19 impact, which may lead to spermatogenic failure.

However, the co-expression of both ACE2 and TMPRSS2 genes was reported only in spermatogonial stem cells and elongated spermatids. The blood-testis barrier (BTB) is not resistant to viruses in its extent. During inflammation, the mumps virus can causes orchitis. So far, 27 viruses have been detected in urine, and 11 in the testis, including the paravaccina virus, the parainfluenza virus, dengue and zika (Atkinson *et al*., 2017). Following LH regulation, the interstitial Leydig cells are responsible for the production of testosterone. Since Leydig and Sertoli cells are covered by blood, the effect of virus on these cells is sufficient to cause infertility, even if the BTB is impermeable to viruses. Another possibility is infection through the accessory glands, the prostate and urinary tracts. Previous studies suggest that many viruses can be actively present and replicate inside the prostate (Spencer *et al*., 2018). Concerning Covid-19, there is evidence that the host cell dipeptidyl peptidase 4 (DPP4) receptor, highly expressed in the prostate, binds to MERS-CoV. We do not have SARS-CoV-2 data yet, but TMPRSS2 is highly expressed in the human prostate epithelium (Table 1), and is androgen-responsive. Therefore, a prostate infection by COVID-19 cannot be ruled out. Clinical data from COVID-19 patients showed SARS-CoV-19 detected in the urine (Wang et al., 2020), as well ACE2 and TMPRSS2, expressed by renal tubular cells (Li *et al*., 2020).

**Table 1:** ACE2 and TMPRSS2 expression levels in human cells of the male urogenital system

	Cells	ACE 2	TMPRSS2	References
Testis	Spermatogonial Stem cells	++++	+++	Shen et al. 2020;
				Stanley et al, 2020;
				Wang et al., 2020
	Differentiated spermatogonia	++	-	Shen et al., 2020; Stanly et al. 2020
	Round spermatids	+	-	
	Elongated spermatids	++	+++	
	Early primary spermatocyte	+	-	
	Spermatozoa	+	-	
	Seminiferous duct cells	+++	-	Stanly et al., 2020; Li et al., 2020
	Leydig cells	+++	Not determined	Shen et al., 2020; Wang et al., 2020
	Sertoli cells	++++	-	Abobaker & Raba, 2020;
				Shen et al., 2020;
				Wang et al., 2020
	Epididymis	+	++	www.proteinatlas.org
Seminal vesicles	Glandular cells	+	+	Shen et al., 2020; Wang et al., 2020
Prostate	Luminal epithelial cells	+	++	
Kidney	Renal Tubular cells	+++	+++	Li et al., 2020
	Glomerular cell	-	-	www.proteinatlas.org
Bladder	Urothelial cells	-	-	www.proteinatlas.org

The mystery of virus in the early fertilization stage remains unknown. There is little evidence-based information about fertilization in the first quarter. Pregnancy outcomes, including birth rate, intrauterine growth restriction and miscarriage, would be useful for making guidelines, but may never support reproduction. Recent clinical reports suggest that the absence of SARS-CoV-2 receptor on spermatozoa, oocytes and embryos, but the myometrium (Goad *et al*., 2020), makes it a low possibility of embryo contamination during IVF treatment. However, at the molecular level, oxidative stress causes activation of pathogenic mechanisms in male fertility, through increases in sperm DNA fragmentation and decreases in progressive motility in the spermatozoa (Agarwal *et al*., 2018; Homa *et al*., 2019). However, increased oxidative stress alter DNA methylation, and affect oocyte performance (Menezo *et al*., 2016); although, in combination with the IVF process, it may suppress the DNA methylation function, with adverse neonatal outcomes (Anifandis *et al*., 2015). A direct effect of this virus on spermatozoa and oocytes/follicles cannot be ruled-out. Yan *et al*. (2013) showed that ACE2 is highly expressed in pre-ovulatory follicles (in rats), human germ cells and early embryos (Honorato-Sampaio *et al*., 2012). One new experimental report shows ACE2 expressed in stromal cells and perivascular cells, which might affect female fertility (Goad *et al*.,2020; Pan *et al*., 2013).

There is a theory that subsequent infertility and testicular damage may result from SARS-CoV-2 infection, and can be sexually transmitted. COVID-19 has been found in the semen of infected patients, but not after recovery (Pan *et al*., 2020). Nevertheless, all available data and scientific findings are recent, based on small sample sizes, limited methodology and show conflicting information. Therefore, until now, there is insufficient scientific data to support that asymptomatic couples should avoid sexual intercourse, as well as those undergoing IVF treatment. There is also a need to find mammary gland cells susceptible to SARS-CoV to overcome the risk of COVID-19 transmission. Moreover, more research is required to understand the long-term effect of SARS-CoV-2 on male/female reproductive function, as well as potential effects on testicular endocrine function and fertility. More detailed physiological and pathological examinations of the male reproductive systems in COVID-19 patients after their recovery are required, because it could be a possible etiopathogenic hypothesis of future infertility, implantation and live birth rates in patients who acquire SARS-CoV-2 infection.
